# Pocket Perforation Using 3-0 Nylon Suture for Device Fixation

**DOI:** 10.7759/cureus.79223

**Published:** 2025-02-18

**Authors:** Daisuke Yamazaki, Mitsunori Yuzurihara

**Affiliations:** 1 Cardiology, Akita Cerebrospinal and Cardiovascular Center, Akita, JPN

**Keywords:** nylon suture, pacemaker pocket infection, pacemaker’s implantation, pocket perforation, thin patients

## Abstract

Device infection occurs at a rate of 1.5%, and age over 65 years is considered a risk factor. If a pacemaker infection occurs, it is recommended that both the generator and pacing lead be removed, which is a highly invasive complication that should be avoided.

We report the case of an 83-year-old man whose 3-0 nylon suture used to fix the generator had perforated the body surface four years after pacemaker implantation. Fortunately, there was no evidence of infection, and thus we were able to repair the perforated portion of the nylon suture and replace the generator without removing the pacing lead. The patient had a body mass index (BMI) of 20.4 kg/m^2^ at the time of pacemaker implantation, but over the course of four years, the patient's BMI had progressively decreased to 18.4. The tip of the nylon suture was sharp, and the thinning of the subcutaneous tissue caused the tip of the nylon suture to perforate the skin. In this case, only the nylon suture protruded through the skin, reminding us of the strong penetrating power of the nylon tip. Preventive measures that are easy for physicians to implement include the use of thin nylon sutures with low penetration force for fixation. It is recommended to use silk sutures when fixing the generator, as the cut ends of the silk sutures are not sharp, and to fix the generator so that a knot is formed at the back. Also, implant a leadless pacemaker. We need to be careful because the number of pacemakers implanted in thin elderly patients is expected to increase in the future.

## Introduction

Device infections occur at a rate of 1.5%, with older patients over 65 considered at risk [1]. Because of the poor blood supply to the area around the device, it is difficult to treat the infection with antibiotics. Therefore, once a pacemaker infection has developed, it is recommended that the generator and leads be removed [2,3]. Once this has occurred, treatment is very invasive. In addition, infective endocarditis and bacteremia are also fatal. For this reason, it is a complication that must be avoided. We report a case in which the 3-0 suture used to fix the pacemaker generator perforated the skin in the remote period, and the generator was replaced before infection occurred. This is a rare case in which only nylon suture perforated the skin in the late stage.

## Case presentation

**Figure 1:**
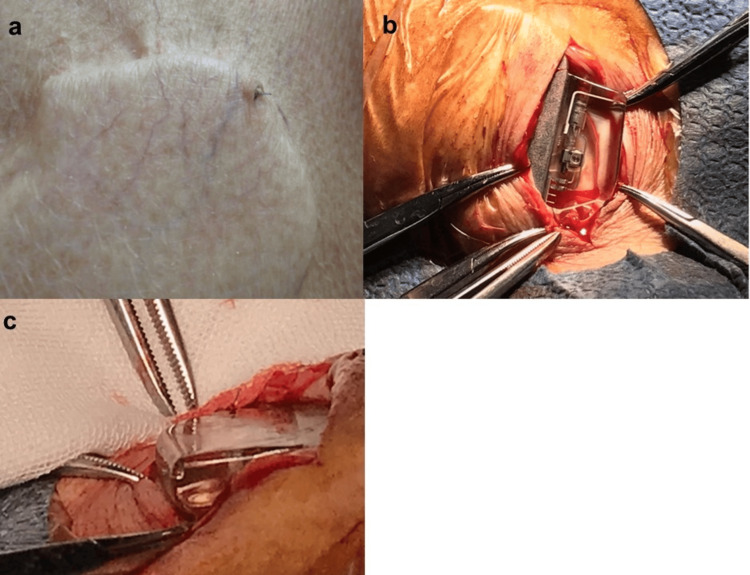
A 3-0 nylon suture perforation and generator replacement. (a) The 3-0 nylon suture used to fix the pacemaker protrudes from the skin. (b) There were no signs of infection around the pacemaker. (c) The tip of the 3-0 nylon suture and the knot can be seen.

An 83-year-old man underwent pacemaker implantation for bradycardia atrial fibrillation complicated with heart failure. The generator used was the Assurity MRI SR generator (Abbott, Green Oaks, IL) and the ventricular lead was the Tendril 58cm lead (Abbott) implanted in the right ventricular septum. A 3-0 nylon suture was used to fix the generator in place. After a two-layer suture with 3-0 absorbable suture, the procedure was completed with a buried suture of the dermis with 5-0 absorbable suture. When the patient was discharged from the hospital, he was 165 cm tall and weighed 55.6 kg. His body mass index (BMI) was 20.4 kg/m^2^. He remained on anticoagulant therapy, attended outpatient clinics, had no recurrence of heart failure, and had his pacemaker parameters checked once a year. However, when the pacemaker was checked four years later, the 3-0 nylon suture was found protruding through the skin (Figure 1a). There was no swelling or redness at the site of the operation, and a scab had formed around the nylon thread. The patient did not have a fever. Blood test showed a white blood cell count of 4,210/μL (reference value: 3,300-8,600/µL), with no change in the neutrophil ratio compared with normal, and no signs of inflammation, with a C-reactive protein level of 0.07 mg/dL (reference value: 0-0.14 mg/dL) (Table 1).

**Table 1:** The blood test results of this case. The patient was thin and had mild hypoalbuminemia and chronic renal dysfunction, but no signs of infection were observed.

Parameters	Results	Reference range	Remarks
White blood cell count	4210 ×10^３^/μL	3.3–8.6^３^/μL	
Neutrophil	67.00%	40.0–70.0 %	
Red blood cell count	360 ×10^4^/μL	430–570 ×10^4^/μL	Low
Hemoglobin	11.7 g/dL	13.5–17.0 g/dL	
Hematocrit	33.90%	40–50 %	Low
Platelet count	6.6 × 10^4^/μL	15–35 × 10^4^/μL	Low
Total protein	6.8 g/dL	6.0–8.0 g/dL	
Albumin	4 g/dL	4.1 -5.1 g/dL	Low
Total bilirubin	1.2 mg/dL	0.4–1.5 mg/dL	
Aspartate aminotransferase	40 U/L	6–40 U/L	
Alanine aminotransferase	23 U/L	6–37 U/L	
Urea nitrogen	37.2 mg/dL	8–20 mg/dL	High
Creatinine	1.45 mg/dL	0.65–1.07 mg/dL	High
Sodium	136 mmol/L	138–145 mmol/L	
Potassium	5 mmol/L	3.6–4.8 mmol/L	High
Chloride	105 mmol/L	101–108 mmol/L	
C-reactive protein	0.07 mg/dL	0–0.14 mg/dL	

The patient weighed 50.2 kg and had a BMI of 18.4 kg/m^2^. Although there was little evidence of pacemaker infection, pacemaker generator replacement was performed because of the risk of future infection. After local anesthesia, the incision was made to pass through the protruding part of the 3-0 nylon suture, and the crusted area was debrided. As shown in Figure 1b, there was no evidence of infection, such as pus or necrotic tissue in the wound, and the 3-0 nylon suture was protruding forward, as shown in Figure 1c. After rinsing with saline, a new generator (Assurity MRI SR) was connected. As a membrane had formed around the generator, the generator was not fixed with sutures, and the procedure was completed by suturing the dermis with 5-0 absorbable sutures after a two-layer suture with 3-0 absorbable sutures. Since then, the patient has progressed well, has shown no signs of infection, and is currently attending outpatient clinics.

## Discussion

Because of the risk of pacemaker infection leading to serious conditions, such as bacteremia and infective endocarditis, it is recommended that the generator and lead be removed completely [2-4]. There is also case report where pacemaker infection was treated conservatively by replacing only the generator, but this is a rare case [5]. However, after a period of time has passed since the pacemaker was implanted, the lead has become adherent to the body, and removal is a very invasive procedure [6]. Therefore, pacemaker infection is one of the complications that must be avoided during pacemaker implantation.

**Figure 2:**
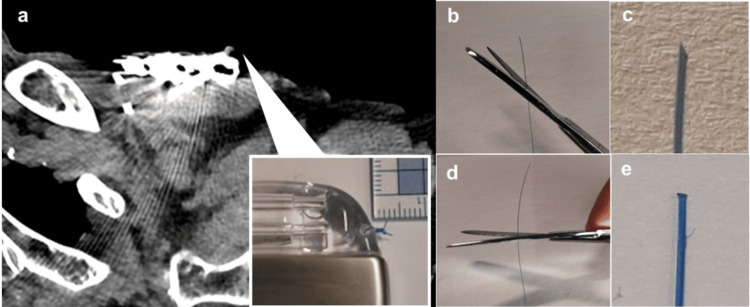
A 3-0 nylon suture knot and cut surface. Figure 2a is an image of this case, and Figures 2b-2e are images created for illustrative purposes. (a) A plain chest CT image when the nylon suture is perforated. The nylon suture knot is formed almost vertically in front of the generator. (b) The 3-0 nylon suture is cut diagonally. (c) Enlarged image of the obliquely cut section. (d) The nylon suture is generally cut horizontally. (e) An enlarged image of the cut end of the horizontal cut.

Older age (>65 years) has been reported to be a risk factor for device infection [1], and hypoalbuminemia has been implicated in device-related pressure injuries [7], which is the case in this case. Although it is occasionally seen that absorbable sutures used to close the dermis of the surgical wound protrude through the skin, there have been no reports of the sutures used to fix the pacemaker protruding through the skin in the long term. The most common type of pocket infection in devices is when the skin becomes fragile, and the generator itself becomes exposed through the skin [8,9]. In this case, it is unusual that only the nylon suture used to fix the pacemaker penetrated the skin and was exposed to the outside. The fact that the suture used to fix the pacemaker is protruding on the surface of the body is equivalent to the generator itself being in contact with the outside, resulting in a high risk of infection. For this reason, the generator was replaced in this case. To reduce the incidence of infection of the suture itself, we use nylon sutures to fix the generator in our institution. Figure 2a shows a plain computed tomography image of a perforated nylon suture. The arrow indicates the knot in the nylon suture. As shown in Figure 2a, if a 3-0 nylon suture is tied twice or three times, a hard knot will form between the generator and the skin, even if it is not sharp, and this is thought to cause irritation to the skin. In addition, the tip of nylon sutures is harder than that of silk sutures. If the nylon suture is cut at an angle as shown in Figure 2b, the tip will become sharp as shown in Figure 2c, and there is a risk of penetration into the skin. In this case, the nylon sutures were cut as shown in Figure 2d. However, when the tip is magnified, as shown in Figure 2e, the edges of the cut nylon sutures are sharp and can cause sufficient irritation to the subcutaneous tissue. It was thought that the knot formed with the 3-0 nylon sutures and the tip of the nylon suture hit the subcutaneous tissue vertically, penetrating the skin over time. In this case, the patient was 83 years old at the time of pacemaker implantation and had a BMI of 20.4 kg/m^2^, and thus there was little subcutaneous fat. However, four years later, since the BMI had dropped to 18.4 kg/m^2^ and the patient had lost even more weight, it is thought that the thinning of the subcutaneous tissue may have been the cause of the nylon suture perforation. The following measures may be considered to prevent perforation when implanting a device in patients with thin subcutaneous tissue: (1) the generator is implanted under the pectoralis major muscle to fix it in place at a deeper level, (2) use thin nylon sutures with low penetration force for fixation, (3) use silk sutures when fixing the generator, as the cut ends of the silk sutures will not be sharp, (4) fix the generator so that a knot is formed at the back, (5) and implant a leadless pacemaker.

Since the implanting of a generator under the pectoralis major muscle requires surgical skill, preventive measures that are easy for cardiologists to perform are considered to be (2) to (5).

The average life expectancy is increasing worldwide, and it is thought that the frequency of implanting devices in elderly, thin patients will increase. When implanting the device in a patient with a thin body shape, it is necessary to perform the procedure so that no stimulation is applied between the generator and the skin, taking into account the possibility that the patient may continue to lose weight even after the implant.

## Conclusions

It is thought that the frequency of implanting devices in thin elderly patients, as in our case, will also increase. Care should be taken when implanting a device in a case with thin subcutaneous tissue to reduce the risk of pacemaker infection. Care should also be taken when using nylon suture to fix the generator, as the knot will be stiff, and even if there are no signs of infection, it is prudent to replace the generator early.
